# Analysis and Respond Surface Methodology Modeling on Property and Performance of Two-Dimensional Gradient Material Laser Cladding on Die-cutting Tool

**DOI:** 10.3390/ma11102052

**Published:** 2018-10-21

**Authors:** Guofu Lian, Mingpu Yao, Yang Zhang, Xu Huang

**Affiliations:** 1 School of Mechanical & Automotive Engineering, Fujian University of Technology, Fuzhou 350118, China; mpyao@smail.fjut.edu.cn (M.Y.); huangxu@fjut.edu.cn (X.H.); 2 School of Engineering + Technology, Western Carolina University, Cullowhee, North Carolina 28723, USA; yzhang@wcu.edu

**Keywords:** laser cladding, two-dimensional gradient material, forming quality, responsive surface methodology

## Abstract

Die-cutting tools have been widely applied in industrial production. However, different forms of failure on a blade, such as wear and fracture, can greatly reduce its service life. In this research, the die-cutting tool was selected as the object, a mixture of high speed steel powder and 304 stainless steel powder was coated as a gradient cladding layer onto the surface of AISI/SAE 1045 steel by laser cladding. The central composition design of the response surface methodology was adopted to establish a mathematical model between the pore area of the multi-layer, multi-track cladding, and its processing parameters: Laser Power (LP), Scanning Speed (SS), Gas Flow (GF), and Overlapping Rate (OR). This model was validated by variance analysis and inspection indicators. The actual experiment value by processing parameters optimization for achieving the smallest pore area showed a 4.41% error compared with the predicted value. The internal structure of the cladding layer is uniform. The defects, such as pores and cracks, meet the requirements. The wear resistance on the cutting edge is about 4.5 times compared with the substrate. The results provide a theoretical guidance for the controlling and prediction of the laser cladding forming quality on a two-dimensional gradient material and the optimization of the processing parameters.

## 1. Introduction

Die-cutting machines have been widely used in printing, packaging, digital accessories for mobile phones, medical supplies, electronic labels, and many other industries. For example, the rotary die cutting machine utilizes the rotation of the rolling die and the continuous feed of the material by the feeding roll to complete the production by die-cutting with the interaction between material and cutting edge. However, the hardness and wear resistance of die-cutting tools restrict the performance improvement of die-cutting equipment. Therefore, the technical bottleneck of the die-cutting machine can be solved by effectively improving cutter body toughness and edge hardness, and enhancing the wear resistance, deformation resistance, and fracture resistance. The current manufacturing technology for cutting edge is usually achieved by milling processing on a high hardness material, such as powder metallurgy high speed steel or a cemented carbide, then followed by heat treatment and grinding. However, large scale cutting tools, such as die-cutting tools, would require stiffness property on its cutting edge to guarantee cutting quality and relatively less internal stiffness to prevent chipping on the cutting edge, which cannot be fulfilled by the aforementioned milling process. Therefore, laser cladding technology can promote the sustainable development of die-cutting machine industries. Current studies on the forming technology of laser cladding have shown that the mechanical performance of the metal cladding layer cannot only meet the requirement of the cutting edge, but also achieve the metallurgical bonding of the materials with different mechanical performance [1].

Laser cladding is an advanced technology used to improve the surface performance of the substrate [2]. Single material cladding is no longer able to satisfy the comprehensive performance requirements. In order to obtain coatings with higher hardness and wear resistance, hard ceramic particles, such as WC, Cr_3_C_2_, TiC, and SiC, are usually added into the melt alloy [3]. However, the different thermal property of the substrate and the added materials tends to cause defects in the cladding layer such as cracks and bad bonding. Consequently, the preparation of a high performance gradient cladding layer requires not only an appropriate preparation method, but also a reasonable material composition design.

Functionally gradient material is a novel material whose composition and structure could provide continuous change as required, thus the property of this material displays a corresponding gradient change [4,5]. Due to this structural feature of functionally gradient material, thermal stress and residual stress generated from the difference of temperature and thermal expansion coefficient could be alleviated, hence prevent the damage [6]. The preparation of functionally gradient material coatings by laser cladding is a new method developed by Jasim et al. [7] in 1990s. The key technology of the preparation process is adjusting the proportion of material components. Lin et al. [8] utilized a two-way powder feeding system to prepare the high-temperature alloy gradient materials of 316L stainless steel and Rene88DT, which achieved the continuous change of stainless steel content varying from 0% to 100%. Li et al. [9] prepared Ni-Cr gradient cladding layer with different mass fractions on the hot die H13 steel, which significantly improved the wear resistance, thermal stability, and fatigue resistance of the substrate. Li et al. [10] prepared composite coatings with different proportions of AlSiTiNi on 304 stainless steel. Studies have shown that with the ratio 50% Al and 20% Ti, the microstructure of the coating is more compact, and the gradient coating has reliable metallurgical bonding with the substrate, which would effectively enhance the hardness and wear resistance of the substrate surface. Weng et al. [11] used Co42 and TiN powder to prepare a gradient cladding layer onto Ti-6Al-4V alloy; this composite coating not only has three to four times microhardness compared with the substrate, but also increases the wear resistance to 18.2 times. Liu et al. [12] successfully prepared a Ni-Ti gradient cladding layer to improve the high temperature and oxidation resistance of the TA2 titanium alloy surface. Studies have found that NiTi, NiTi2, and Ni3Ti produced in a gradient cladding layer could significantly enhance the microhardness and oxidation resistance.

Regarding the performance of gradient material cladding layer, Zhou et al. [13] investigated the influence of laser remelting on the structure and performance of the gradient cladding layer prepared with WC powder and a Fe-based amorphous alloy powder. Their research found that remelting could reduce the crack and porosity, and effectively improve the quality of cladding layer. Amado et al. [14] studied the crack issue in two-layer WC gradient material preparation. They found that the 60% wt WC cladding layer would not generate cracks even if the substrate was not preheated when 15% wt WC content as the intermediate layer. This indicated that the gradient change of the material composition could be an effective solution to the crack problem. Bartkowski et al. [15] studied the effects of gradient materials on the microhardness and corrosion resistance of the cladding layer through preparing Stellite-6/WC gradient coating. They found that the microhardness and corrosion resistance of the cladding layer were significantly improved by adding different proportions of WC powder. Lin et al. [16] researched the microstructure and mechanical performance of TiB2/TiB gradient layer, found that the microhardness and elastic modulus gradually decreased from the coating surface to the substrate, while the cracks and toughness showed a gradual increase.

In the study of the effects on the performance of gradient materials resulting from the processing parameters, Weng et al. [17] prepared Co42/B4C gradient materials onto a Ti-6Al-4V substrate and analyzed the microstructure and wear resistance of the cladding layer under three variable processing parameters: laser specific energy, laser power, and scanning speed. The results showed that the cladding layer with a lower laser specific energy could obtain higher microhardness and wear resistance. Mahamood et al. [18] prepared TiC/Ti6Al4V gradient materials onto Ti-6Al-4V substrate and studied the effects of scanning speed on the microstructure, microhardness, and wear resistance of the cladding layer. It was found that the cladding layer satisfying the performance requirement could be obtained by adjusting the scanning speed. Kamran et al. [19] probed into the effects of the processing parameters on the gradient structure of Steel-Ni. Specifically, they investigated the effects of processing parameters on the microstructure, hardness, and wear resistance of the cladding layer with continuous changing laser power and powder feeding rate, while maintaining constant conditions such as feed rate, airflow velocity, and substrate temperature.

At present, studies on functionally gradient materials are still in the initial stage both in material design and preparation process. In the study Bartkowski et al. conducted, only two WC percentage (30% and 60%) composite sample coating of Stellite-6/WC was prepared by laser cladding, which only provides technical reference on a one-dimensional gradient material [15]. Zhang et al. selected different volume ratio composite of Ti6Al4V and TiC as cladding layer, but they were also restricted to a one-dimensional gradient coating [20]. Majumdar et al. designed one-dimensional gradient Fe/SiC layer with 85 wt % Fe + 15 wt % SiC as top layer and 95 wt % Fe + 5 wt % SiC as bottom layer cladded onto mild steel [21]. It can be seen that research had been conducted on a one-dimensional gradient material, but the performance of two-dimensional gradient cladding layer has rarely been discovered, where the two-dimensional gradient cladding material remains unchanged along the Z-axis, but possesses continuous changes along the X-axis and Y-axis due to the selection of different materials and different powder mixture ratios. Thus, a two-dimensional gradient cladding layer could be achieved with the appropriate cladding material selection to fulfill the requirement of different stiffness on the cutting edge and the internal for ensuring the cutting quality and to prevent chipping on the cutting edge. According to the current research on laser cladding, the mechanical property of the cladding layer could accomplish the aforementioned expectation, and also implement proper metallurgical bonding between materials with different mechanical properties [1,22]. However, the cladding layer quality, such as pore area and crack potential is the critical problem in the application phase. Li et al. investigated a one-dimensional gradient coating of Ti/TiBCN on a 7075 aluminum alloy substrate with a TiBCN weight percentage 0%, 5%, 10%, and 15%, but noticeable cracks and pores could be observed in the coating [23]. Wang et al. discovered that the forming of a crack during cladding was related to the thermal property of the cladding layer material and substrate, such as the thermal expansion coefficient and Young’s modulus. Selection of a similar thermal property for the cladding material and substrate could reduce the defects in cladding layer [24]. Shi et al. pointed out that the composite gradient coating could diminish the defects and benefit the property of the cladding layer [25]. Therefore, the lack of research in a two-dimensional gradient material cladding layer promotes the object of this study as the laser cladding manufacturing of die cutting tools with two-dimensional continuous hardness gradient material of a cladding layer onto the substrate, hence the pore area in the cladding layer is selected to be the quality evaluation criteria. The coupling effect of different processing parameters on the pore area of the multi-layer and multi-track cladding layer was analyzed by a central composite design method. The discovery could provide a theoretical basis for laser cladding manufacturing of large size cutting tools.

## 2. Materials and Methods 

**Figure 1:**
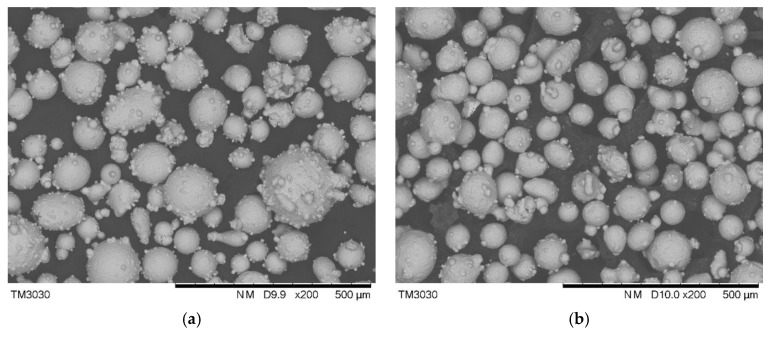
(**a**) Morphology of high speed steel powder; (**b**) Morphology of 304 stainless steel powder.

**Figure 2:**
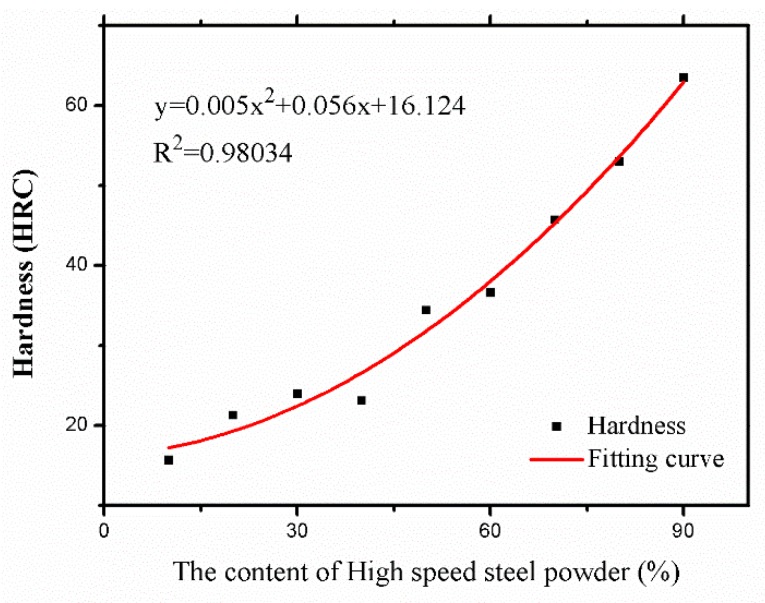
Polynomial fitting curve.

**Figure 3:**
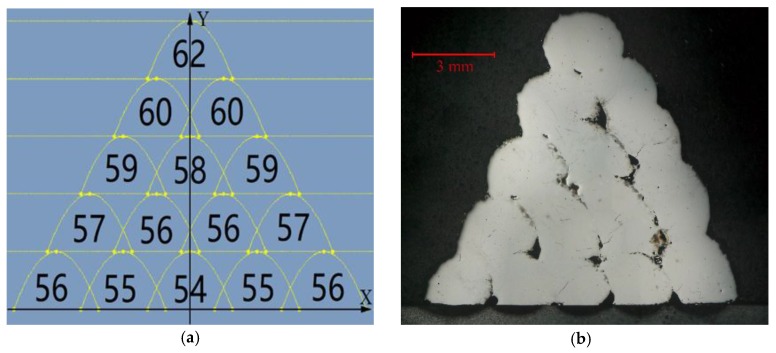
(**a**) Hardness gradient distribution; (**b**) Two-dimensional hardness gradient cladding layer.

The substrate utilized in this experiment was AISI/SAE 1045 steel (50 mm × 50 mm × 10 mm), since it is a medium carbon quenched and tempered steel with high quality and low price. The two types of laser cladding powder selection were high speed steel powder (W6Mo5Cr4V2) and 304 stainless steel powder with a particle size of 48 μm–106 μm, produced by Chengdu Huayin Powder Technology CO., LTD (Chengdu, China). Since high speed steel powder (W6Mo5Cr4V2) processes have outstanding thermoplastic properties and wear resistance, it is usually selected as the material for the cutting tool [26]. 304 stainless steel is resistant to high temperatures up to 800 °C when its hardness as a cutting edge is satisfied to fulfill the cutting job. It maintains excellent toughness and is easy to machine, so it is selected to accomplish the requirement of avoiding chipping from the internal cutting tool [27]. A proper ratio of a two powder mixture could complete the favorable property and bonding with the substrate. The morphology of these two types of powder is illustrated in Figure 1. Their corresponding composition is shown in Table 1. Before laser cladding, the substrate was cleaned with acetone to remove the oil stain, then rinsed with alcohol and dried afterwards. Nine groups of high speed steel powder with content varying from 10% to 90% were mixed with stainless steel powder at the same powder feeding rate. These nine groups of powder mixture were dried in a vacuum dryer at 120 °C for 30 min. YLS-3000 fiber laser system (IPG, Burbach, Germany)was used to prepare a multi-layer and multi-track cladding layer. The following parameters were selected by the preliminary experiment: laser power 1.4 kW, scanning speed 7 mm/s, gas flow 1000 L/h, and defocusing amount (distance between substrate surface to laser beam focus) 6 mm. Argon was filled as the protective gas during the cladding process. After cladding, the samples were cut and inlaid into metallographic pattern. Then they were grinded with metallographic sandpaper from coarse to fine, and etched in aqua regia for 30 s, and dried by cold air after rinsing. The microhardness of the cladding layer was tested with a MVA-402TS microhardness tester (HDNS, Shanghai, China), which measured the hardness data as shown in Figure 2. The relationship between the composition of the cutting tool material and its corresponding hardness was established with the Origin software. The fitted curve in Figure 2 could provide a reference based on the different hardness requirements for the corresponding powder mixture composition. Figure 2 illustrates that the fitting curve R^2^ = 0.98034 has a high degree of fitness, from which the powder ratio could be calculated according to the required hardness. When the hardness is 62HRC, the life of a die cutting tool is about completing 3 million pieces of designated products such as diapers or bandage from nonwoven fabric, according to the data provided by Sanming PNV Machinery Co., Ltd. (Sanming, China). Therefore, the top cladding layer hardness was selected as 62HRC. Figure 3a shows the two-dimensional hardness gradient material distribution of the cutting edge, designed as five cladding layers. The first layer was designed with similar hardness as 54–56 HRC to that of the heat-affected zone, where is the transition area between the substrate and cladding layer, to improve the adhesion between the cladding layer and the substrate. The hardness of the cladding layer showed a gradual increase both from the origin to the positive or negative direction on the X-axis and from the origin to the positive direction on the Y-axis. Two-dimensional hardness gradient cladding layer corresponding to the shape of the cutting edge was prepared with the same processing parameters, shown in Figure 3b, where the continuous two-dimensional hardness gradient material was achieved. However, porous defects were also established in its internal structure. Figure 3b reveals that many cracks were developed at pores where the stress was concentrated.

Response Surface Methodology (RSM) is an optimization method for comprehensive experimental design and mathematical modeling, which can get explicit functional relationships between the influence factors and the target with fewer experiments [28]. The effects of laser power, scanning speed, gas flow rate, and overlap ratio on the pore area of the gradient cladding layer was revealed by a response surface methodology, which helped to improve the performance of the two-dimensional gradient cladding layer by reducing the pore area. The pore area could be derived by Equation (1) by measuring the area of the cladding layer, where A_p_ stands for pore area, A stands for cladding layer area, P_p_ stands for pixels in the pore area, and P stands for pixels in the cladding area. Since the area could be determined by pixel, and pixels could be obtained by the processing of gray-level transformations, fuzzy image enhancement, and binary segmentation on the image [29]. The RSM central composite design of 30 experiment sets and their corresponding results were shown in Table A1. Later, the validation was conducted on the sample developed with the processing parameters after optimization. Scanning electron microscope (SEM) observation on the structure of the cladding layer was conducted by TM3030PLUS (HITACHI 550I, Tokyo, Japan). The wear resistance of the cladding layer was tested by UMT-2 high load scratch tester. In the wear resistance testing experiment, a tungsten steel ball with a diameter of 4 mm was selected as the grinding head material, with a 10 mm friction distance, 20 minutes of testing time, 20 N grinding head load, and 15 mm/s sliding speed.

(1) $$\frac{A_{p}}{A}=\frac{P_{p}}{P}\;$$

## 3. Results and Discussion 

### 3.1. Effects of Processing Parameters on Pore Area

By conducting a significance analysis on the response model’s coefficient and lack of a fit, the second order regression model was selected for the pore area. The analysis of variance for the pore area is presented in Table A2. P-value (prob > F) indicates the probability when the event that occurred is larger than F, which here stands for the significance of model coefficient. The less significant factors were eliminated by a stepwise regression. The selected model has the value of prob > F less than 0.01%, and the value of lack of fit’s prob > F is larger than 0.05, meaning that the probability the model would be distorted by the interference factors is only 0.01%. Therefore, this model has remarkable fitting accuracy and the input variables have significant influence on the response value. The selection of the model is reasonable. The value of adequate precision is 26.383, greatly larger than 4. This means that the model’s accuracy could meet the requirements, making it highly recognizable. A larger multi-factor coefficient R^2^ (0 ≤ R^2^ ≤ 1) displays a better correlation. Values of R^2^, adjusted R^2^, and predicted R^2^ are all near to 1. The difference between the adjusted R^2^ and predicted R^2^ is less than 0.2, so this model maintains enough accuracy. From Table A2, each processing parameter has a different level of influence on the pore area, among which the scanning speed, overlapping rate, interaction between laser power with scanning speed, gas flow, and overlapping rate, interaction between scanning speed with gas flow and overlapping rate, and the respective square of laser power and gas flow all have a significant influence on the pore area. By central composition design, the regression coefficient for each model could be obtained. The fitted model equation for pore area (A_p_) is listed in Equation (2).

(2) $$A_{p}=422.000-279.785\cdot LP+1.692\cdot SS-0.330\cdot GF-3.138\cdot OR-8.633\cdot LP\cdot SS+0.132\cdot LP\cdot GF+1.173\cdot LP\cdot OR+5.594e^{-3}\cdot SS\cdot GF+0.206\cdot SS\cdot OR+56.001\cdot LP^{2}+4.886e^{-5}\cdot GF^{2}\;$$

**Figure 4:**
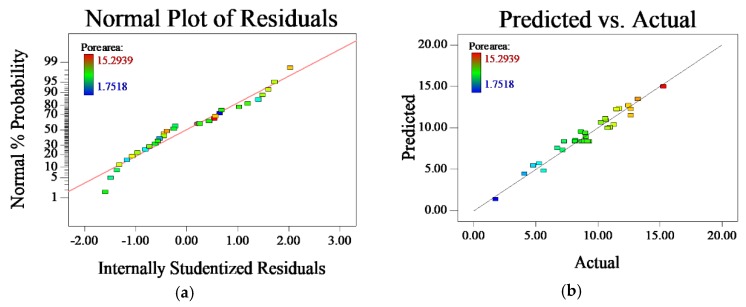
(**a**) Residual analysis of pore area; (**b**) Comparison plot between predicted and actual pore area

Figure 4a is the normal distribution plot of residuals for the pore area. The level of the input parameter is listed as the X-axis and Y-axis exhibit normal probability. It can be seen that the residuals are almost normally distributed on the same straight line, indicating that the model fits well. Figure 4b is the comparison between the predicted and actual values. Actual value is listed as the colored dots, predicted value is projected as the straight line, a close distance indicates relatively small difference between actual and predicted value. The small deviation between the two values reveals the high accuracy of the model’s prediction.

**Figure 5:**
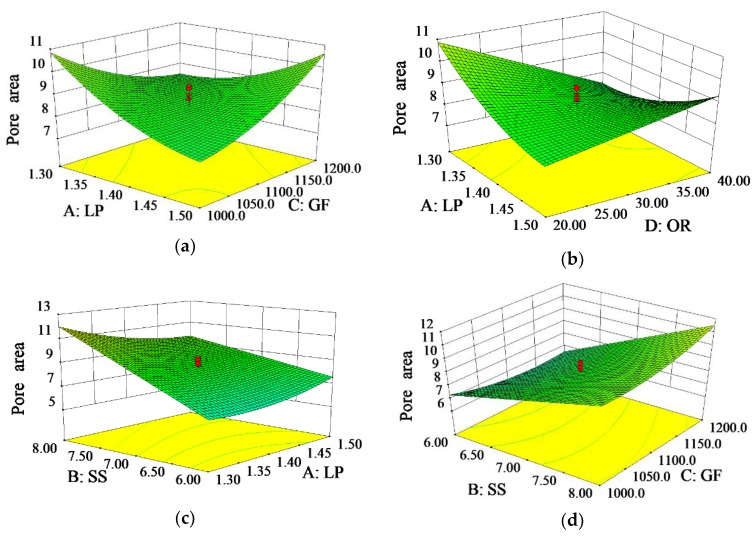

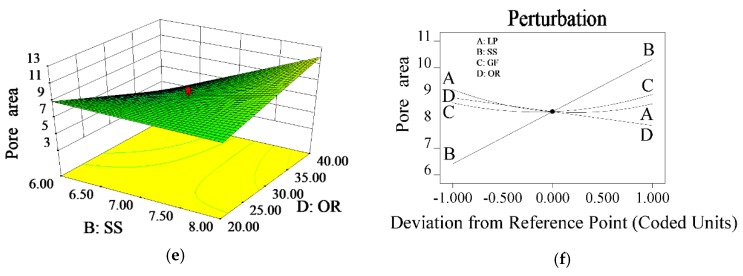
(**a**) 3D influence curve of pore area by interaction of LP and GF; (**b**) 3D influence curve of pore area by interaction of LP and OR; (**c**) 3D influence curve of pore area by interaction of SS and LP; (**d**) 3D influence curve of pore area by interaction of SS and GF; (**e**) 3D influence curve of pore area by interaction of SS and OR; (**f**) Influence curve of pore area from different parameters.

Figure 5a shows the effects of the interaction between the laser power and the gas flow on the pore area. It illustrates that the pore area tends to increase when the laser power and gas flow increase or decrease at the same time. As shown in Figure 5b, a lower laser power and a lower overlapping rate result in an increase in pore area. In Figure 5c, the pore area is rising with the decreasing of the laser power and increasing of the scanning speed. Figure 5d indicates that the pore area increases when scanning speed and gas flow increase simultaneously. Figure 5e shows an increase of the pore area with an increase of the scanning speed and overlapping rate. Figure 5f presents a diagram of the effects of various factors on pore area, within which the scanning speed is the most significant factor. The pore area tends to rapidly increase with a larger scanning speed. Instead, it shows that the overlapping rate increment would lead to a reduction on the pore area, which is also a significant factor. Similarly, the pore area tends to decrease with the increase of the laser power, but it displays a decrease first and then an increase with the increasing gas flow.

The substrate absorbs less energy with decreasing laser power, which leads to a weakening convection of the molten pool. Then the pores do not have enough time to overflow, resulting in a larger pore area. The decrease of laser power will lead to a decreased amount of molten powder. Meanwhile, a lower overlapping rate will cause not only void defects when the same layer is overlapped, but also leads to the collapse of the upper cladding layer due to the unevenness created during the multiple lower cladding layers overlapping. When the laser power decreases, the solidification rate of the cladding layer increases at a higher scanning speed. The pores do not have enough time to overflow, resulting in a raise of pore area [30,31]. With the increase in the scanning speed, the laser beam stays a shorter time on the surface of the substrate and the cladding layer [28]. Consequently, less energy is absorbed when the cladding layer is stacked onto the substrate, and the duration of the molten pool becomes shorter. Meanwhile, too much powder enters the pool with the increase of air flow, worsening the fluidity of molten pool, which causes an increase to the pore area [32]. When the scanning speed and overlapping rate increase simultaneously, the surface of the cladding layer becomes uneven because of the higher overlapping rate. Therefore, more pores will be generated during the stacking process, as the defect rate between the layers greatly increases.

### 3.2. Optimization on Processing Parameters and Model Validation

**Figure 6:**
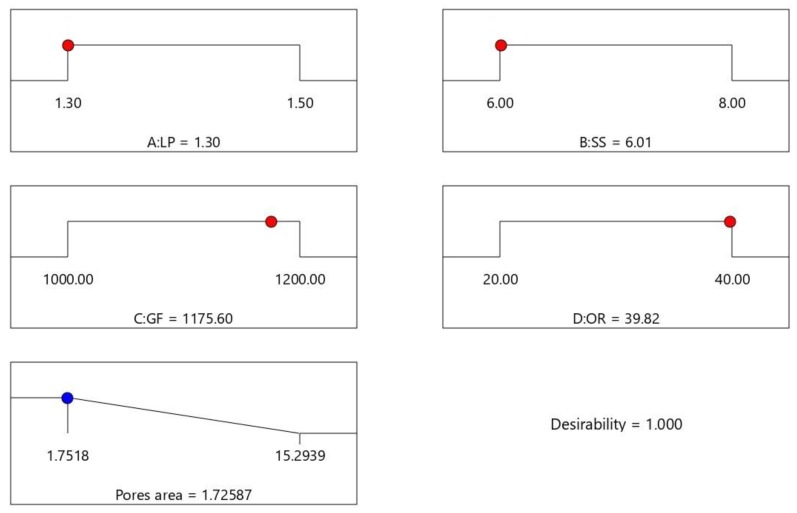
Optimization results of processing parameters and expected optimal pore area.

**Figure 7:**
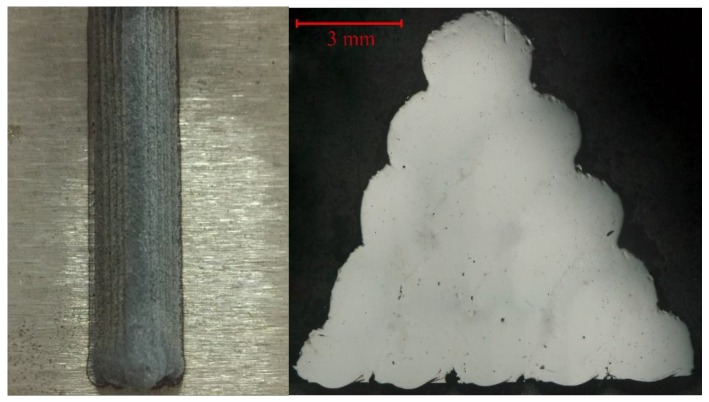
Geometrical characteristics of two-dimensional gradient cladding (**left**) and cross-section of multi-track cladding layer (**right**).

Table 2 lists the optimization criteria and target for the processing parameters and response. Processing parameters are set as “in range” according to the preliminary study that the acceptable cladding layer could be achieved within those ranges [28]. The minimum pore area is set as the optimization goal. The weight value of the pore area is selected as 5, which is the maximum weight value that can be assigned. All other weight values are assigned as the default weight value 3. Optimization results are shown in Figure 6, where the red dots stand for the numerically optimal parameter position in the range of processing parameters setting and the blue dot stands for the optimal response value in the prediction. A desirability value of 1 indicates all the criteria have been met and the optimal result could be expected under these optimal processing parameters. The set of processing parameters with the highest expected value is listed as LP = 1.3 kW, SS = 6.01 mm/s, GF = 1175.60 L/h, OR = 39.82%, and predicted P_A_ = 1.726 mm^2^. An experiment was conducted with 1.3kW as the laser power, 6mm/s as the scanning speed, 1175 L/h as the gas flow, and 39.82% as the overlapping rate, where the aforementioned parameters were acquired within the equipment capability and close to the optimal parameters. The laser cladding results of this experiment are shown in Figure 7, which displays the cladding layer’s appearance, cross-section morphology, and pore area, which reveals that the layer surface is relatively smooth with fewer pores. The actual measured value of the pore area is 1.802 mm^2^, with an error rate of 4.41%, which meets the requirement of the error rate within 5% under the accuracy of the equipment. Therefore, the established model has remarkable prediction accuracy and provides significant guidance for controlling pore area in a multi-layer and multi-track laser cladding, as well as improving the performance of the cladding layer. 

## 4. Performance of cladding layer 

### 4.1. Structure of the Cladding Layer

**Figure 8:**
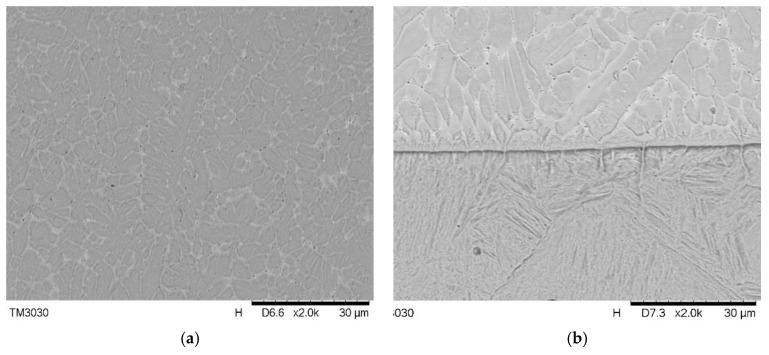
(**a**) Cladding layer structure; (**b**) Interface between substrate and cladding layer.

A metallurgical microstructure analysis was conducted on the cutting edge’s cladding layer, and the interface between the cladding layer and the substrate. Figure 8a presents the cladding layer’s microstructure at the cutting edge. Through laser cladding, this unbalanced rapid solidification procedure, the grain size in cladding layer kept relatively uniform, in which the majority displayed as an isometric crystal with a size ranging from 5–6 μm and some grown to a columnar crystal. No noticeable pore or crack that may weaken the mechanical property was observed under 2000× magnification. As a comparison, the cladding layer and substrate interface are shown in Figure 8b. The different morphology was formed due to the different thermal progress that resulted from a high power laser thermal input on the top and the rapid heat radiation process of the metal substrate’s good heat transfer property [33]. Most of the grains in the interface area were dendrite and columnar crystals, whose grain size was significantly increased. The area of stress concentration would establish performance variation interfaces; defects or even cracks could be easily formed under rapid temperature change in the laser cladding [34]. The interface shown in Figure 8b displays a good metallurgical bonding at the interface, where no obvious pore or fracture that could diminish the mechanical property was observed under 2000× magnification. Thus, a high quality multi-layer and multi-track laser cladding coating without defect is achieved with processing parameters generated from the mathematical model. 

### 4.2. The Wear Resistance of the Cladding Layer

**Figure 9:**
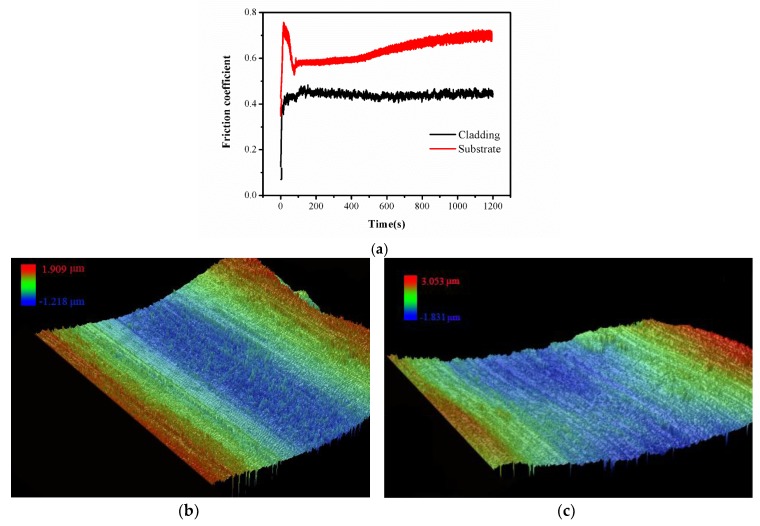
(**a**) Friction coefficient curve of top cladding layer and substrate; (**b**) Wear on the cladding layer; (**c**) Wear on the substrate.

Figure 9a is the friction coefficient curve obtained from the wear resistance test by a UMT-2 high load scratch tester of the cutting edge’s cladding layer and the substrate comparison varying with time. This figure is derived from the sample test with maximum expected value, when LP was 1.3 kW, SS was 6 mm/s, GF was 1175 L/h, and OR was 39.82%. The substrate’s friction coefficient stabled around 0.54 initially; as the friction test proceeded and the friction deepened there was a larger area of spherical friction in contact with the AISI/SAE 1045 steel substrate, and the friction coefficient increased to 0.72. Comparatively, the friction coefficient of the cladding layer had less fluctuation amplitude, which stabled between 0.41 and 0.44. Compared with AISI/SAE 1045 steel, the friction coefficient of the cladding layer reduced dramatically. The wear volume was calculated by measuring the 3D morphology with white light interferometry on wear area and shown in Figure 9b,c. The wear volume of the cutting edge’s cladding layer was 2.29 × 10^−3^ mm^3^, while that of the substrate was 1.03 × 10^−2^ mm^3^. Under the same experimental parameters, the volume wear had reduced by nearly 80%; in other words, the wear resistance was enhanced 4.5 times compared with an AISI/SAE 1045 steel substrate.

## 5. Conclusions

The die-cutting tool was taken as the research object and central composition design response surface methodology was utilized to develop a regression model between laser cladding processing parameters and results. This model revealed the inherent functional relationships between the pore area of the multi-layer, multi-track cladding layer and laser power, scanning speed, gas flow, and overlapping rate. The reliability of the model was verified by the experiments. The macroscopic and microstructure and wear resistance of the cladding layer were analyzed comprehensively. The results provide a reference for an optimal processing parameter selection and quality control of the gradient cladding layer. The conclusions can be drawn as follows:

1. The interaction between the laser power and gas flow has a major influence on the pore area. The cladding layer with a smaller pore area can be obtained by increasing the laser power while decreasing the gas flow. The scanning speed also has a notable influence on the pore area of the cladding layer. The pore area of the cladding layer can be reduced by decreasing the scanning speed and gas flow or by increasing the overlapping rate and decreasing the scanning speed properly. Under low laser power, the pore area can be decreased by increasing the overlapping rate or by decreasing the scanning speed.
2. By selecting the optimal processing parameters, the cladding layer could have a minimum pore area, uniform internal structure, fewer pores and cracks. Therefore, the model developed in this research displays significant guiding purpose for the control and prediction of quality and the internal structure in multi-layer multi-track cladding layers.
3. The wear resistance of the cutting edge cladding layer shows about 4.5 times than that of the substrate, which indicates the tool’s wear resistance can be remarkably improved by the laser cladding of two-dimensional gradient materials.

## Figures and Tables

**Table 1:** Powder elements composition (wt %) of high speed steel (W6Mo5Cr4V2) and 304 stainless steel (304L).

Material	Element (wt %)								
	Si	Mn	C	V	Cr	Mo	W	Ni	Fe
W6Mo5Cr4V2	0.15–0.4	0.2–0.45	0.8–0.9	1.75–2.2	3.8–4.4	4.5–5.5	5.5–6.75	-	Rest
304L	0.28	4.16	0.029	-	19.19	-	-	11.26	Rest

**Table 2:** Optimization Criteria.

Variables or Response	Criterion	Limit		Importance
		Lower	Upper	
LP	in range	1.2	1.6	3
SS	in range	5	9	3
GF	in range	800	1200	3
OR	in range	10	50	3
Pore Area	Minimum	0.018	0.187	5

## Appendix A

**Table A1:** Central composition design and results.

Run Order	LP (kW)	SS (mm/s)	GF (L/h)	OR (%)	Pore Area (mm^2^)
1	1.5	8	1200	20	8.642
2	1.4	7	900	30	10.801
3	1.3	6	1200	40	1.752
4	1.4	7	1300	30	10.236
5	1.4	7	1100	30	9.163
6	1.5	8	1000	20	4.803
7	1.5	6	1000	20	8.193
8	1.4	9	1100	30	12.657
9	1.4	7	1100	30	9.293
10	1.4	7	1100	30	7.293
11	1.5	6	1200	20	11.294
12	1.4	7	1100	50	7.169
13	1.3	8	1200	20	12.657
14	1.4	7	1100	30	8.704
15	1.2	7	1100	30	10.612
16	1.4	7	1100	10	8.994
17	1.3	6	1200	20	9.035
18	1.5	8	1000	40	10.654
19	1.4	7	1100	30	8.163
20	1.3	8	1200	40	11.507
21	1.3	8	1000	40	13.250
22	1.6	7	1100	30	10.996
23	1.5	6	1000	40	5.268
24	1.5	8	1200	40	15.294
25	1.4	5	1100	30	4.086
26	1.5	6	1200	40	6.724
27	1.3	6	1000	20	11.736
28	1.3	8	1000	20	12.469
29	1.3	6	1000	40	5.634
30	1.4	7	1100	30	8.894

**Table A2:** Analysis of variance for pore area in response surface quadratic model.

Source	Sum of Squares	Mean Square	F Value	p-Value Prob > F	
Model	249.118	22.647	34.079	<0.0001	significant
B	91.189	91.189	137.220	<0.0001	
D	6.405	6.405	9.639	0.0061	
AB	11.925	11.925	17.944	0.0005	
AC	28.020	28.020	42.164	<0.0001	
AD	22.001	22.001	33.107	<0.0001	
BC	5.007	5.007	7.535	0.0133	
BD	68.115	68.115	102.499	<0.0001	
A^2^	8.921	8.921	13.424	0.0018	
C^2^	6.790	6.790	10.218	0.0050	
Lack of Fit	9.170	0.705	1.263	0.4258	not significant
Adeq Precision	26.383	R-Squared		0.9542	
Pred R-Squared	0.8386	Adj R-Squared		0.9262	

A: LP, B: SS, C: GF, D: OR, AB: Interaction between LP and SS, etc.
